# Behavioural evidence for separate mechanisms of audiovisual temporal binding as a function of leading sensory modality

**DOI:** 10.1111/ejn.13242

**Published:** 2016-04-08

**Authors:** Roberto Cecere, Joachim Gross, Gregor Thut

**Affiliations:** ^1^ Centre for Cognitive Neuroimaging (CCNi)Institute of Neuroscience and PsychologyUniversity of Glasgow58 Hillhead StreetG12 8QBGlasgowUK

**Keywords:** multisensory integration, simultaneity judgments, temporal binding window, temporal processing

## Abstract

The ability to integrate auditory and visual information is critical for effective perception and interaction with the environment, and is thought to be abnormal in some clinical populations. Several studies have investigated the time window over which audiovisual events are integrated, also called the temporal binding window, and revealed asymmetries depending on the order of audiovisual input (i.e. the leading sense). When judging audiovisual simultaneity, the binding window appears narrower and non‐malleable for auditory‐leading stimulus pairs and wider and trainable for visual‐leading pairs. Here we specifically examined the level of independence of binding mechanisms when auditory‐before‐visual vs. visual‐before‐auditory input is bound. Three groups of healthy participants practiced audiovisual simultaneity detection with feedback, selectively training on auditory‐leading stimulus pairs (group 1), visual‐leading stimulus pairs (group 2) or both (group 3). Subsequently, we tested for learning transfer (crossover) from trained stimulus pairs to non‐trained pairs with opposite audiovisual input. Our data confirmed the known asymmetry in size and trainability for auditory–visual vs. visual–auditory binding windows. More importantly, practicing one type of audiovisual integration (e.g. auditory–visual) did not affect the other type (e.g. visual–auditory), even if trainable by within‐condition practice. Together, these results provide crucial evidence that audiovisual temporal binding for auditory‐leading vs. visual‐leading stimulus pairs are independent, possibly tapping into different circuits for audiovisual integration due to engagement of different multisensory sampling mechanisms depending on leading sense. Our results have implications for informing the study of multisensory interactions in healthy participants and clinical populations with dysfunctional multisensory integration.

## Introduction

Most events in the environment tap into different sensory systems at the same time. For any such event, optimal integration of its multisensory properties into one coherent percept can improve perception (Sumby & Pollack, 1954; Stein & Meredith, 1993; Vroomen & de Gelder, 2000; Ramos‐Estebanez *et al*., 2007; Kim *et al*., 2012; Cecere *et al*., 2014) and behaviour (Gielen *et al*., 1983; Hughes *et al*., 1994; Colonius & Diederich, 2004; Romei *et al*., 2007).

Previous research has shown that temporal proximity of inputs is an important factor promoting multisensory integration (e.g., Meredith *et al*., 1987) and increases the likelihood that multiple sensory cues are attributed to a common external source (Welch, 1999; Spence, 2007). However, multisensory integration based on the timing of neuronal input is not trivial to achieve, as temporal discrepancies exist across modalities in both physical transmission and neural processing speed (Harris *et al*., 2008). It has therefore been suggested that, for proper binding of multisensory information, the brain needs to tolerate small time lags. The window of tolerance to asynchrony, i.e. in which two asynchronous stimuli are judged synchronous, is called the temporal binding window (TBW; Colonius & Diederich, 2004).

A great deal of research has focused on perception of audiovisual simultaneity, capitalizing on behavioural paradigms such as simultaneity judgment (SJ) and temporal order judgment (TOJ) tasks using different measures of perceived simultaneity such as the TBW as well as the point of subjective simultaneity (PSS; for a review, see Keetels & Vroomen, 2012). These studies showed that perception of simultaneity varies across a number of factors, including individual differences (Stevenson *et al*., 2012), developmental stages (Hillock *et al*., 2011), tasks/stimulus features (van Eijk *et al*., 2008; Stevenson & Wallace, 2013; Leone & McCourt, 2015) and attended sensory modality (Zampini *et al*., 2005; Spence & Parise, 2010). Studies specifically focusing on the plasticity of simultaneity judgments reported temporal recalibration effects (i.e. shifts in PSS) after exposure to fixed audiovisual time lags (Fujisaki *et al*., 2004; Vroomen *et al*., 2004) and changes in sensitivity to asynchrony (i.e. TBW narrowing) following perceptual training (Powers *et al*., 2009, 2012). In addition to evidence for plasticity, many studies point to consistent biases in synchrony perception. For instance, audiovisual pairs tend to be judged more synchronous when the visual stimulus leads (bias in PSS; for a review, see Keetels & Vroomen, 2012) and sensitivity to asynchrony is usually higher for auditory‐leading (auditory–visual; AV) as compared to visual‐leading (visual–auditory; VA) pairs (e.g., Dixon & Spitz, 1980; Conrey & Pisoni, 2006; van Wassenhove *et al*., 2007), resulting in a narrower TBW for AV than VA pairs (TBW asymmetry). Finally, TBW asymmetries have also been pointed out by studies investigating audiovisual binding from a developmental perspective, reporting much earlier maturation of the VA TBW than the AV TBW (e.g., Hillock *et al*., 2011).

Despite consistent reports of asymmetries in audiovisual binding depending on the leading sense, little is known about the underlying mechanisms. One possible account is that natural differences in audiovisual latencies (light travels faster than sound, leading to more frequent exposure to VA than AV pairings) have forged the PSS bias and that the TBW asymmetry is a consequence thereof. Indeed, with a PSS bias in favour of VA synchrony perception, the entire psychometric curve of audiovisual simultaneity judgments would shift towards VA pairs, consequently narrowing the AV and widening the VA window. Note that extensive experience with the more natural visual‐leading stimuli is also thought to forge the developmental bias in favour of the VA TBW (e.g., Hillock *et al*., 2011). Alternatively, the asymmetry in the TBW may suggest that temporal binding of audiovisual information is not a unitary process but involves two distinct mechanisms of multisensory interactions depending on the leading sense (see van Wassenhove, 2013; Thorne & Debener, 2014). By extension, different mechanisms underlying AV and VA TBW may then also contribute to differences in their developmental timeline. However, to date it has not been possible to distinguish between these two alternative accounts, as independence of AV and VA integration has not been systematically addressed.

In the present behavioural study, we capitalised on the plasticity of the TBW to test whether a single (unitary) vs. a dual (modality‐specific) mechanism regulates audiovisual temporal binding in simultaneity judgments. To this end, we employed a training paradigm by Powers *et al*. (2009) that has proven to enhance the accuracy of simultaneity judgments, and which we modified to examine whether training is transferable between audiovisual pairs (AV vs. VA). Three groups of participants performed an audiovisual SJ task before and after 2 days of feedback‐based perceptual training, practicing respectively on both AV and VA pairs, VA pairs only or AV pairs only. Through this manipulation we sought to test to what extent selective training with one stimulus pair (e.g. AV or VA) influenced the non‐trained stimulus pair (VA or AV, respectively). In case in which binding of AV and VA pairs is governed by distinct mechanisms, one would predict absence of crossover after AV‐only and VA‐only training.

## Materials and methods

### Participants

Thirty‐four healthy volunteers gave written informed consent to participate in this study, which was approved by the Ethics Committee of the College of Science and Engineering, University of Glasgow, UK. All participants had normal or corrected‐to‐normal vision and normal hearing by self‐report. Four participants were excluded because of inconsistent behavioural performance in pre‐tests which did not fit a sigmoidal curve (see Data analysis). To assess the effects of different training protocols on SJs, the remaining 30 participants were split into three groups of 10 participants each: (i) AV+VA training group (mean age, 21.5 years; seven female; nine right‐handed), receiving SJ training with both auditory‐ and visual‐leading audiovisual pairs (AV and VA intermixed); (ii) VA‐only training group (mean age, 22 years; three female; nine right‐handed), receiving SJ training with visual‐leading pairs only; (iii) AV‐only training group (mean age, 21.2 years; five female; eight right‐handed), receiving SJ training with auditory‐leading pairs only.

### Experimental design

**Figure 1:**
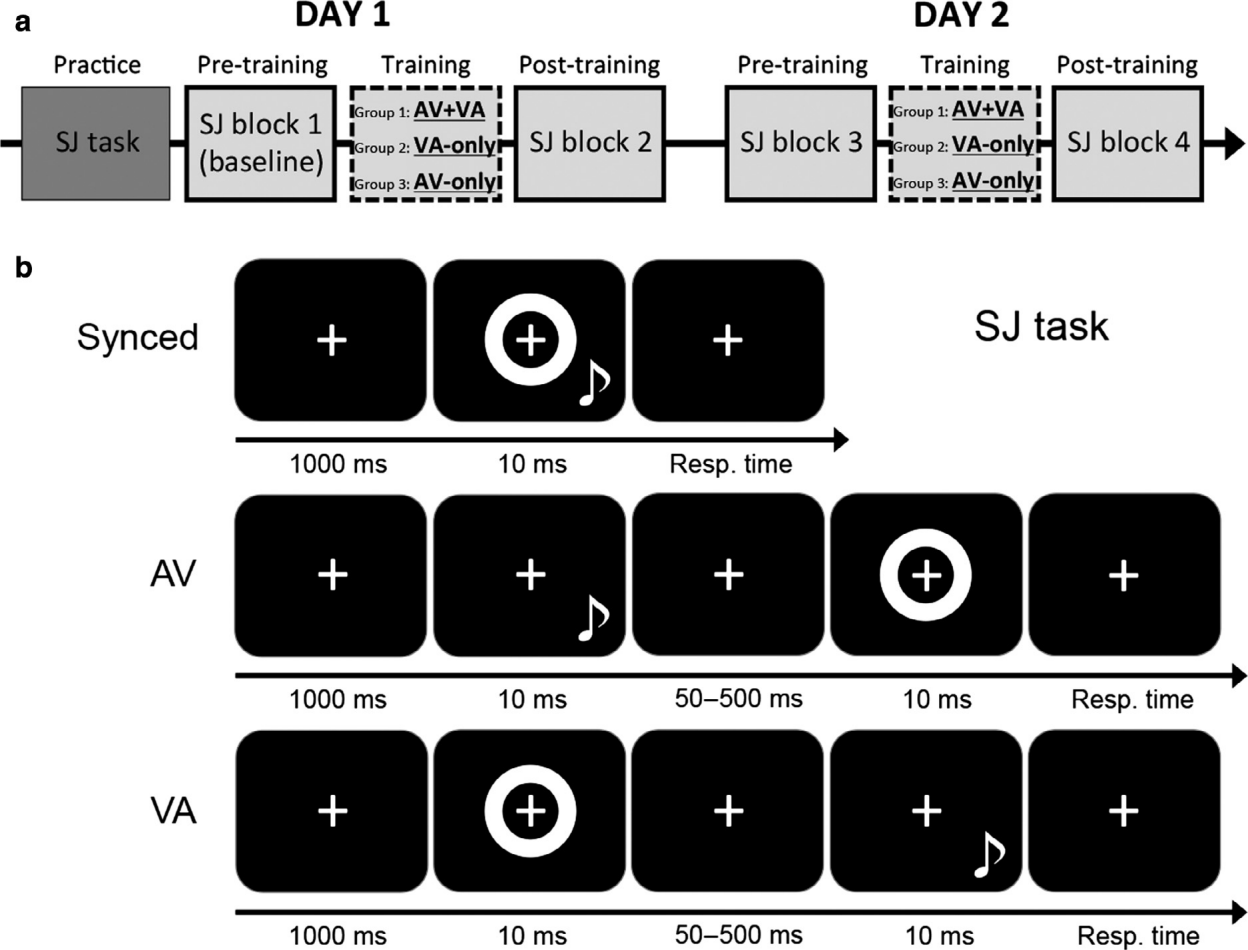
Schematic representation of (a) the experimental protocol and (b) the trial structure of the simultaneity judgment task.

The experimental procedure was modified from Powers *et al*. (2009). Each group of participants underwent two experimental sessions over two consecutive days (see Fig. 1a). On day 1, participants first performed a 5‐min practice session for familiarization with the SJ task (represented in Fig. 1b). This was followed by: (i) an SJ block without feedback to assess the sensitivity to asynchrony at baseline for AV and VA stimulus pairs (pre‐training block; see Simultaneity judgment assessment), (ii) the training block consisting of SJ with feedback using either mixed AV+VA, VA‐only or AV‐only stimulus pairs (see Simultaneity judgment training) and (iii) another SJ block without feedback to reassess sensitivity for AV and VA pairs (post‐training block). The session on day 2 was identical to day 1, except for the absence of the practice session. We regarded the pre‐training SJ block on day 1 (labelled SJ task 1) as the baseline, and the other three SJ blocks (labelled SJ tasks 2–4) as post‐training follow‐ups when analysing the data.

### Simultaneity judgment assessment

In the behavioural task, participants evaluated simultaneity of audiovisual stimulus pairs in which the auditory and visual components could be presented with different inter‐stimulus delays. The visual stimulus was a white annulus (outer diameter, 9 visual degrees; inner diameter, 4.5 visual degrees) that was flashed around the fixation point for 10 ms (Fig. 1b). The auditory stimulus consisted of a 10‐ms sinusoidal pure tone (frequency, 1800 Hz; sampling rate, 44 100 Hz) delivered via a loud‐speaker (sound pressure level, ~ 75 dB) positioned at the bottom of the monitor and vertically aligned to the visual stimulus. A central fixation cross (1 × 1 visual degrees) was continuously displayed on a black background for the entire duration of the SJ blocks.

In each trial, after displaying the fixation cross for 1000 ms, both the visual and auditory stimuli were presented either simultaneously or temporally misaligned, with one out of 13 possible stimulus onset asynchronies (SOAs): 0 ms (synchronous condition); − 500, − 250, − 200, − 150, − 100, − 50 ms (asynchronous conditions, auditory stimulus leading; AV); + 50, + 100, + 150, + 200, + 250, + 500 ms (asynchronous conditions, visual stimulus leading; VA). Participants responded by pressing button 1 on the keyboard for ‘synchronous’ and button 2 for ‘asynchronous’. A new trial started immediately after the response. Each of the four SJ blocks (pre‐/post‐training, day 1/day 2) consisted of 260 trials (13 conditions × 20 repetitions) presented in 2 × 130 trials with a short break in between.

### Simultaneity judgment training

For training, the same stimuli and task as for SJ assessments were used, but a subset of onset asynchronies were presented (individually adjusted), feedback was given after each trial, and mixed AV+VA, VA‐only or AV‐only stimulus pairs were presented depending on training group.

The trial structure (identical for all the three training groups) consisted of a fixation cross presented for 1000 ms, followed by the audiovisual stimulus pair (presented either synchronously or asynchronously). After the participant's response, visual feedback was presented for 500 ms in the centre of the screen in the form of the word ‘correct!’ (green font) or ‘incorrect!’ (red font).

Experimental conditions differed between training groups. For the AV‐only group, receiving a selective SJ training for auditory‐leading pairs, stimuli were presented at four possible SOAs, i.e. either synchronously (SOA, 0 ms) or with the sound leading the visual flash by three different SOAs (individually adjusted to equate training difficulty across participants, see below). Similarly, for the VA‐only group, receiving a selective SJ training for visual‐first pairs, stimuli were presented either synchronously (SOA, 0 ms) or with the visual flash leading the sound by three SOAs (again, individually determined). Finally, for the AV+VA group receiving a training that included both AV and VA pairs, there were seven possible SOAs, one synchronous (SOA, 0 ms) and six asynchronous (three AV SOAs and three VA SOAs, also individually adjusted).

For the training blocks only, SOAs were individually tailored to each participant in order to adjust training/task difficulty across participants and across the AV‐ and VA‐TBWs which typically show individual differences (e.g., Stevenson *et al*., 2012). Avoiding the use of identical SOAs for all participants specifically aimed to ensure that a potential training benefit is equated across conditions (AV and VA pairs) and individuals (no benefit expected if too easy). Note that this contrasts with Powers *et al*. (2009, 2012) who used a similar training protocol but with fixed SOAs (± 50, ± 100, ± 150 ms), probably differing in training difficulty across participants and conditions (more difficult for less sensitive participants and for VA pairs than for AV pairs, as TBW width is typically 200–250 ms for VA pairs and 100–150 ms for AV pairs). Here, we first estimated the size of each participant's TBW, separately for AV and VA pairs, based on the performance in the pre‐training SJ blocks. This was done by fitting two sigmoid functions (AV pairs, [*y* = 1/(1 + exp(−(*x* − *a*)/*b*))]; VA pairs, [*y *=* *1/(1 + exp(−(*a* − *x*)/*b*))]) to the behavioural data using the MATLAB Curve Fitting Toolbox (The MathWorks Inc., Natick, MA, USA). The *x* value (in ms) of the sigmoid corresponding to *y *=* *75% probability of perceived simultaneity (*x*_75%_) was considered the individual width of the TBW. The three SOAs selected for training were respectively 1/3 × *x*_75%_, 2/3 × *x*_75%_, and *x*_75%_, thus obtaining three equidistant SOAs all falling within the individual TBW.

AV‐only and VA‐only training consisted of 20 repetitions × 3 asynchronous SOAs plus 60 repetitions of the 0‐ms SOA (240 AV‐only or VA‐only training trials respectively, presented in 2 × 120 trials with a break in between). A higher number of repetitions for the 0‐ms SOA was used to balance the likelihood of occurrence of synchronous and asynchronous conditions (see also Powers *et al*., 2009). For the same reason, the AV+VA training group performed 20 repetitions × 6 asynchronous SOAs plus 120 repetitions of the 0‐ms SOA (240 AV training trials and 240 VA training trials, also presented in two blocks). Training SOAs were randomly intermixed.

Note that with this design, the number of training trials per AV and VA pair was equated across the training groups (i.e. AV training trials = 240 in both AV‐only and AV+VA training groups, and VA training trials = 240 in both VA‐only and AV+VA training groups). This ensured that each side of the curve (AV or VA temporal binding windows) received the same amount of training per group, allowing for a direct comparison of each TBW (AV or VA) between groups.

### Apparatus

During the experimental sessions participants sat on a comfortable chair with their head placed on a chin rest at 57 cm distance from a CRT monitor (100 Hz refresh rate). Stimulus presentation was controlled by a computer running e‐prime software (Version 2.0). Responses were collected using a standard keyboard.

### Data analysis

**Figure 2:**
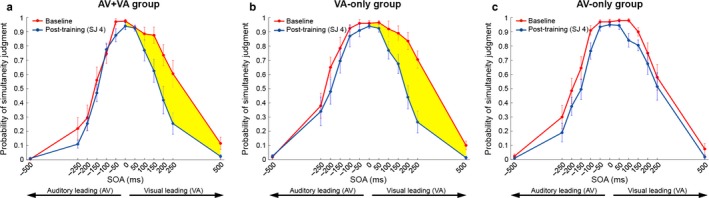
Average probability of perceiving audiovisual synchrony (*y*‐axis) as a function of SOA (*x*‐axis), before (red lines) and after simultaneity judgment training (i.e. SJ session 4; blue lines) with (a) AV+VA pairs, (b) VA‐only pairs and (c) AV‐only pairs. Error bars represent SEM. Yellow areas highlight improvement in detecting audiovisual simultaneity, which was evident for visual‐leading conditions after AV+VA and VA training, but not after AV training.

Probability scores were visually inspected (Fig. 2) but statistical analyses were performed on *d*‐prime (*d*′) scores which were calculated for each of the asynchronous SOAs (≠ 0 ms; noise) with respect to the synchronous SOA (= 0 ms; signal) to index sensitivity to asynchrony and to control for possible response bias induced by the training (i.e. changes in the overall response pattern that could be mistaken for genuine changes in synchrony perception). anovas were used to first evaluate performance at baseline as a function of leading sense. To this end, we conducted a 3 × 2 × 6 mixed‐design anova on baseline data with Training Group (AV+VA training, VA‐only training, AV‐only training) as between‐subjects factor and Leading Sense (auditory‐leading, visual‐leading) and SOA (50, 100, 150, 200, 250, 500 ms) as within‐subject factors. Second, we examined whether different types of SJ training (AV+VA, VA‐only, AV‐only) had a selective effect on the trained pairs only (i.e. no crossover) or a global effect on simultaneity judgments of both the trained and untrained pairs. To this end, we performed an omnibus 3 × 2 × 4 × 6 mixed‐design anova with the additional within‐subject factor SJ Session (four levels: baseline, 2, 3, 4). All significant effects and interactions were further broken down by simple tests and followed up by Bonferroni *post hoc* tests where appropriate. Huynh–Feldt corrections were applied to compensate for violations of sphericity. Partial eta‐squared ($\eta_{p}^{2}$) and Cohen's *d* were used to evaluate effect sizes.

## Results

**Figure 3:**
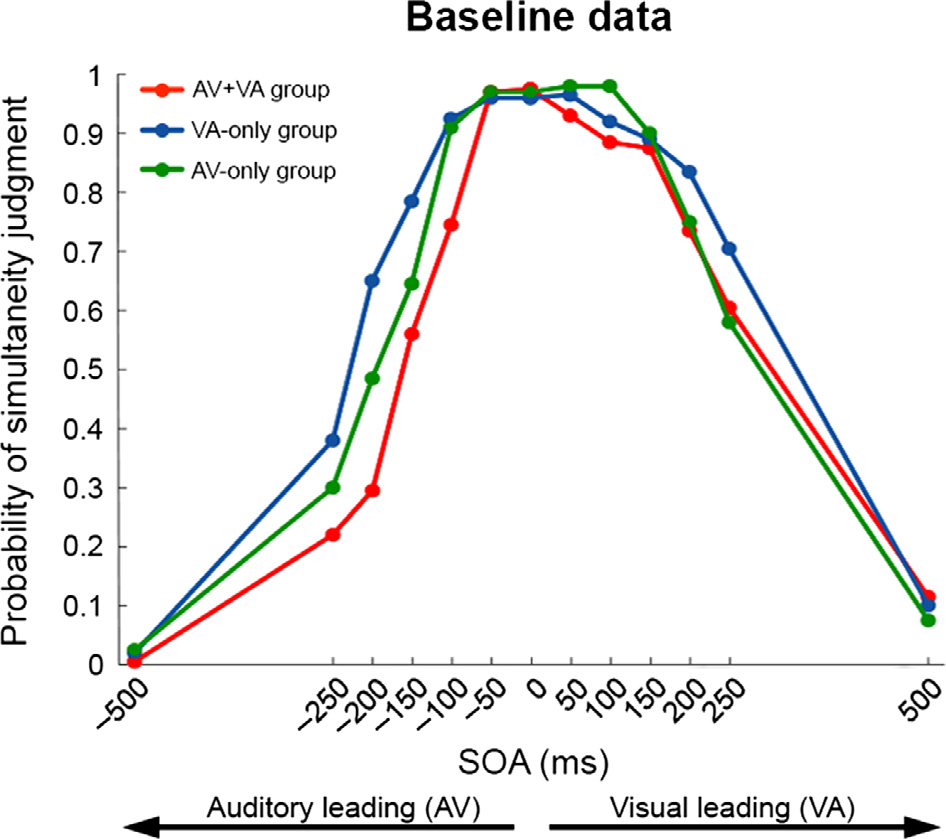
Superimposed plots of AV+VA group's (red), VA‐only group's (blue) and AV‐only group's (green) pre‐training (baseline) performance in the simultaneity judgment task.

Figure 2 shows raw behavioural data of each training group in the SJ task before (SJ block 1, baseline) and after (SJ block 4) receiving specific SJ training. Considering baseline only (red curves), an asymmetry in the ability to detect audiovisual asynchrony is evident as a function of leading sense (Fig. 2a–c; compare left vs. right side of red curves). For all groups, red curves appear narrower for auditory‐leading conditions (left side) than for visual‐leading conditions (right side), reflecting higher accuracy in detecting asynchrony for auditory‐leading trials. Considering trainability of simultaneity judgments (baseline vs. post‐training; red vs. blue curves), the data revealed training benefits (narrowing of curves) to be present only on the right side of the curves (visual‐leading conditions; Fig. 2a and b, yellow areas), whereas the left side of the curves (auditory‐leading conditions) never improved with training. Notably, training benefits for visual‐leading conditions were only evident when training included VA stimulus pairs, i.e. in the AV+VA training group (Fig. 2a) and the VA‐only training group (Fig. 2b) but not the AV‐only training group (Fig. 2c). Therefore, regarding transferability, detection of audiovisual asynchrony did not improve in the trainable, visual‐leading conditions when training involved auditory‐leading pairs (no crossover effects; see Fig. 2c). Importantly, these group differences in training outcome for visual‐leading conditions cannot be explained by differences in baseline performance, as baseline curves overlapped for the three training groups (see Fig. 3, right side of curves).

These three main findings regarding asymmetry, trainability and transferability between the TBWs were statistically confirmed as detailed below.

### Asymmetry in TBW at baseline as a function of leading sense

**Figure 4:**
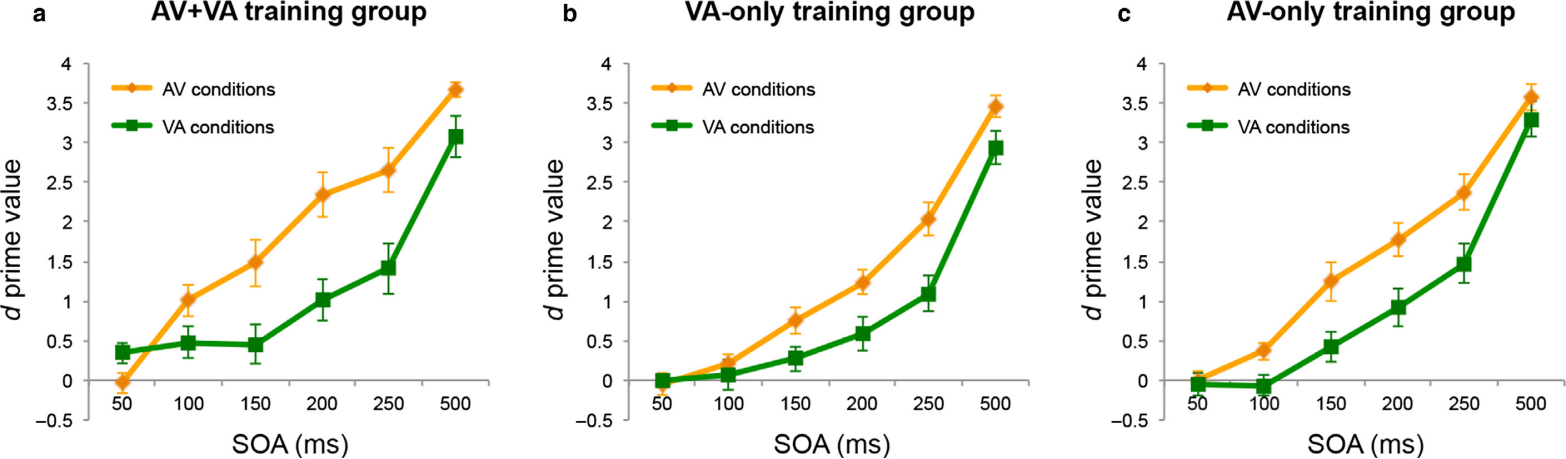
Sensitivity to asynchrony (*d*′, *y*‐axis) in the pre‐training SJ session (baseline) during simultaneity judgment of AV or VA pairs (AV vs. VA conditions) as a function of SOA (*x*‐axis), shown separately for the three groups [(a) AV+VA, (b) VA‐only and (c) AV‐only training]. For all groups, overall sensitivity to asynchrony was higher (i.e. narrower TBW) for AV stimulus pairs (orange lines) than for VA stimulus pairs (green lines).

A Training group × Leading sense anova on baseline (SJ1) *d*′ scores showed that before training (Fig. 4), sensitivity to asynchrony in audiovisual simultaneity judgment depended on the Leading Sense (main effect of Leading Sense, *F*_1,27_ = 37.101, *P *<* *0.0001, $\eta_{p}^{2}$ = 0.58), independently of Training Group (*F*_2,27_ = 0.7; *P* = 0.5, $\eta_{p}^{2}$ = 0.05) (Fig. 4a vs. b vs. c). In line with previous findings (e.g., Dixon & Spitz, 1980; Conrey & Pisoni, 2006; van Eijk *et al*., 2008), participants showed overall higher sensitivity to asynchrony (i.e. higher *d*′ values) in auditory‐first conditions (*d*′ = 1.58) than in visual‐first conditions (*d*′ = 0.98; Fig. 4a–c, orange vs. green lines). *Post hoc* planned comparison between overall *d*′ scores in AV and VA TBWs confirmed the AV‐VA asymmetry separately for each group (all *P *<* *0.01).

### Differences in trainability of TBWs for auditory‐leading vs. visual‐leading stimulus pairs

**Figure 5:**
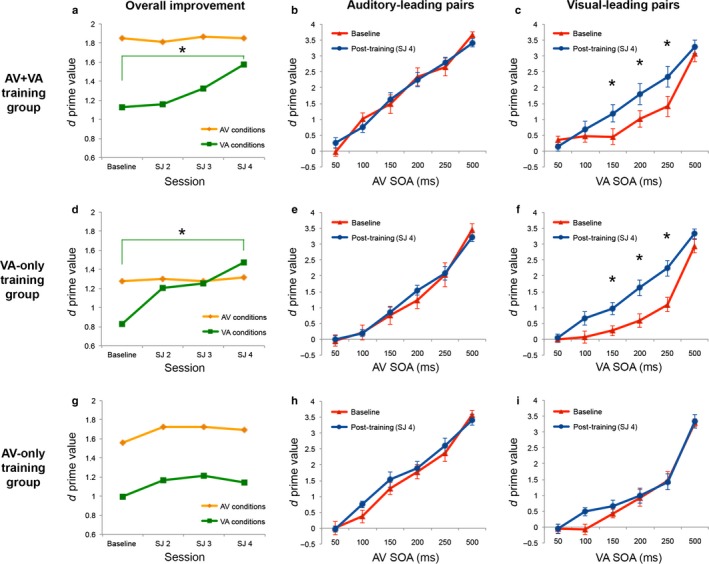
Sensitivity scores (*d*′) for AV+VA (upper row), VA‐only (middle row) and AV‐only (lower row) training groups. The leftmost column (a, d and g) shows, for each training group, the overall change in sensitivity to asynchrony (i.e. collapsed across SOAs) related to training (i.e. over sessions), separately for AV (yellow lines) and VA (green lines) stimulus pairs. The middle and rightmost columns show, separately for AV (b, e and h) and VA (c, f and i) pairs the *d*′ change from baseline (red lines) to the last post‐training assessment (blue lines) at each SOA. *P <* *0.02 for *d*′ changes after training.

Trainability of audiovisual simultaneity judgments for auditory‐ and visual‐leading pairs was examined first for the AV+VA training group (Fig. 5a–c), before disentangling the specific contributions of VA‐only vs. AV‐only training (Fig. 5d–i, see section below). Breaking down the analysis by Training group was statistically justified by the omnibus 3 × 4 × 2 × 6 anova revealing a significant three‐way interaction of Training Group × SJ Session × Leading Sense (*F*_6,81_ = 3.50, *P* = 0.005, $\eta_{p}^{2}$ = 0.21) and a significant four‐way interaction of Training Group × SJ Session × Leading Sense × SOA (*F*_30,405_ = 1.51, *P* = 0.045, $\eta_{p}^{2}$ = 0.1).

AV+VA training (Fig. 5a–c) improved sensitivity to audiovisual asynchrony (*d*′) across SJ sessions (effect of training) depending on Leading Sense (two‐way interaction SJ Session × Leading Sense; *F*_3,27_ = 5.65, *P *=* *0.013, $\eta_{p}^{2}$ = 0.39; Fig. 5a). Unpacking this interaction revealed that AV+VA training progressively increased *d*′ for VA pairs over SJ blocks (Fig. 5a, green line, main effect of SJ Session; *F*_3,27_ = 4.48, *P *=* *0.01, $\eta_{p}^{2}$ = 0.33), driven by improved performance in the last post‐training SJ block as compared to baseline (SJ block 1 vs. 4: mean *d*′ = 1.13 vs. 1.57, *P *=* *0.018, Cohen's *d *=* *1.11; Fig. 5a). Sensitivity for AV pairs remained unchanged after training (Fig. 5a, orange line; *F*_3,27_ = 0.07, *P *=* *0.98, $\eta_{p}^{2}$ = 0.008).

In addition, AV+VA training improved *d*′ depending on SOA and Leading Sense (three‐way interaction SJ Session × Leading Sense × SOA: *F*_15,135_ = 3.37, *P *<* *0.0001, $\eta_{p}^{2}$ = 0.27; see Fig. 5b and c). Separate analyses for each Leading Sense showed, for VA pairs (Fig. 5c), an SJ Session × SOA interaction (*F*_15,135_ = 4.1, *P *<* *0.0001, $\eta_{p}^{2}$ = 0.31) which was driven by improved sensitivity after training (SJ4) at the 150‐ms (*d*′ = 1.19), 200‐ms (*d*′ = 1.8) and 250‐ms (*d*′ = 2.35) SOAs, as compared to baseline (150‐ms SOA: *d*′ = 0.45; 200‐ms SOA: *d*′ = 1.01; 250‐ms SOA: *d*′ = 1.41; all *P *<* *0.001, all Cohen's *d *≥* *0.83; Fig. 5c). For AV pairs (Fig. 5b), there was a trend for SJ Session × SOA interaction (*F*_15,135_ = 1.8, *P *=* *0.06, $\eta_{p}^{2}$ = 0.17), driven by changes across sessions between different SOAs. However, when specifically looking at the contrasts that are relevant to our analysis (i.e. how *d*′ at a given SOA changed over sessions), there was no significant change for any SOA (all *P* = 1).

The above findings reveal asymmetries in audiovisual integration as a function of leading sense in terms of both TBW width (expressed as overall *d*′) at baseline and its trainability (expressed as *d*′ change), confirming results from Powers *et al*. (2009). However, employing a mixed training protocol (AV+VA) does not make it possible to disentangle the contribution of each sub‐component of training (AV and VA) to the observed TBW changes and hence to rule out potential crossover effects (i.e. training of AV conditions influencing simultaneity judgments for VA pairs). In other words, with such a mixed protocol it is not possible to establish whether a common or two independent mechanisms regulate AV and VA temporal binding.

### Trainability and transferability of simultaneity judgments after AV‐only and VA‐only training

To test for a potential independence of binding mechanisms for auditory‐ and visual‐leading stimulus pairs, we investigated the effects of selective SJ training (i.e. VA‐only or AV‐only) on sensitivity to asynchrony for the untrained pair.

VA‐only training (Fig. 5d–f) led to a similar pattern as AV+VA training (cf. Fig. 5a–c). It increased *d*′ over SJ sessions depending on Leading Sense (two‐way interaction SJ Session × Leading Sense; *F*_3,27_ = 10.78, *P *<* *0.0001, $\eta_{p}^{2}$ = 0.55; Fig. 5d). Separate analyses for each Leading Sense showed that VA‐only training progressively increased *d*′ for VA stimulus pairs over SJ blocks (Fig. 5d, green line; main effect of SJ session, *F*_3,27_ = 6.08, *P *=* *0.003, $\eta_{p}^{2}$ = 0.4), with a significant sensitivity improvement from baseline to post‐training (SJ1 vs. SJ4: mean *d*′ = 0.83 vs. 1.48, *P *=* *0.0016, Cohen's *d *=* *1.69; Fig. 5d). In contrast, sensitivity for AV pairs remained unchanged (Fig. 5d, orange line; *F*_3,27_ = 0.02, *P *=* *0.99, $\eta_{p}^{2}$ = 0.003).

In addition, similarly to AV+VA training, we observed a trend for three‐way interaction of SJ Session × Leading Sense × SOA (*F*_15,135_ = 1.65, *P *=* *0.069; Fig. 5e and f). Unpacking this interaction revealed an SJ Session × SOA interaction for VA stimulus pairs (Fig. 5f; *F*_15,135_ = 2.73, *P *=* *0.001, $\eta_{p}^{2}$ = 0.23), explained by a *d*′ increase for the 150‐ms (*d*′ = 0.97), 200‐ms (*d*′ = 1.63) and 250‐ms SOAs (*d*′ = 2.24) as compared to baseline (150‐ms SOA, *d*′ = 0.27; 200‐ms SOA, *d*′ = 0.59; 250‐ms SOA, *d*′ = 1.1; all *P *<* *0.017, all *d *≥* *1.27). No such effects were evident for AV pairs (no SJ Session × SOA interaction: *F*_15,135_ = 1.04, *P* = 0.42, $\eta_{p}^{2}$ = 0.1; Fig. 5e).

In contrast to the AV+VA and VA‐only training groups, there was no evidence of training effects in the AV‐only group (Fig. 5g–i). AV‐only training did not affect *d*′ over SJ blocks (no main effect of SJ blocks: *F*_3,27_ = 1.05, *P* = 0.39, $\eta_{p}^{2}$ = 0.1) and there was also no interaction with Leading Sense (no two‐way interaction SJ Session × Leading Sense: *F*_3,27_ = 0.11, *P *=* *0.96, $\eta_{p}^{2}$ = 0.01; Fig. 5g) or SOA (no three‐way interaction SJ Session × Leading Sense × SOA: *F*_15,135_ = 1.11, *P *=* *0.35, $\eta_{p}^{2}$ = 0.1; Fig. 5h and i). Crucially, this negative result suggests that practicing simultaneity judgments of AV pairs not only failed to improve sensitivity to asynchrony for (trained) AV conditions but, more importantly, did not affect the judgment of (untrained) VA conditions, which, on the other hand, was clearly responsive to within‐condition training (VA and AV+VA training).

Nevertheless, in light of the finding by Powers *et al*. (2009) that the amount of improvement after training depends on the initial (baseline) size of the TBW, it is important to ensure that the lack of improvement in the VA conditions after AV‐only training was genuine and not driven by baseline differences in sensitivity to asynchrony between groups, e.g. the AV‐only group already being at ceiling at baseline. Our analysis of baseline performance (see above: *Asymmetry in TBW at baseline as a function of leading sense*) had already revealed a consistent asymmetry in sensitivity to asynchrony (AV TBW < VA TBW) across all training groups (see Training group × Leading sense anova and *post hoc* comparisons), and no Training group × Leading sense interaction. However, to further rule out baseline differences across groups as a potential confound, we run additional *post hoc* tests comparing groups within each AV and VA TBW, none of which proved significant (AV TBW, all *P *>* *0.19; VA TBW, all *P *=* *1). Hence, none of our reported differential training effects on the right‐side of the (VA) temporal binding window (or the absence thereof) can be explained by differences at baseline.

Taken together, the above results demonstrate that there is no crossover between AV and VA conditions, i.e. that evaluating audiovisual simultaneity involves separate mechanisms for auditory‐leading and visual‐leading pairs.

## Discussion

Previous research using a variety of tasks and stimuli to investigate temporal audiovisual integration has shown that humans display a certain degree of tolerance to audiovisual asynchrony and that such tolerance is typically greater for asynchronous stimulus pairs where visual stimulus leads (VA) compared to those where auditory stimulus leads (AV) (e.g., Conrey & Pisoni, 2006; van Eijk *et al*., 2008; Stevenson & Wallace, 2013). Notwithstanding these observations, auditory‐before‐visual and visual‐before‐auditory integration may be regarded as part of a continuum, implying that the AV and VA TBWs are two sides of the same coin, with perception of audiovisual simultaneity being biased towards visual‐leading stimulus presentation (see Keetels & Vroomen, 2012 for a review). Our data suggest an alternative scenario, demonstrating that simultaneity judgments of AV and VA pairs are empirically separable based on at least three aspects (asymmetry in TBW width, trainability, and, most importantly, non‐transferability). Collectively, this therefore indicates that AV and VA temporal integration are independent and may be underpinned by separate rather than one single mechanism.

Our experimental design is based on the idea that if audiovisual temporal integration is governed by different mechanisms as a function of leading sense, we should find not only dissociations with regard to asymmetries in TBW width and trainability (e.g., Powers *et al*., 2009) but crucially also reveal absence of crossover, i.e. training one type of judgment (e.g. auditory‐leading pairs) should not transfer to the non‐trained type of judgment (e.g. visual‐leading pairs). Regarding trainability, our data revealed that whereas sensitivity to asynchrony improved in visual‐leading conditions (narrower VA‐TBW) as a consequence of training with visual‐leading pairs (AV+VA and VA‐only), auditory‐leading conditions were not sensitive to feedback (unchanged AV‐TBW) after both AV+VA and AV‐only training. This is consistent with previous work (Powers *et al*., 2009, 2012) showing no changes in AV judgments after feedback‐based audiovisual training. However, compared to Powers *et al*. (2009), our study more firmly establishes non‐trainability of AV judgments because using training regimes that have been individually adjusted for task difficulty (not controlled for previously) and because we introduced a selective training condition (specifically training AV‐only pairs) to the previously used compound training protocol (AV+VA). Our data clearly suggest that sensitivity to asynchrony for auditory‐leading pairs is already at ceiling and/or governed by efficient and less malleable mechanisms, at variance with perception of VA asynchrony which appears to be less accurate but more flexible (i.e. trainable). Regarding transferability, we found that training one type of audiovisual pairs (e.g. auditory‐leading) did not cross over to the other type (e.g. visual‐leading). We note that the lack of transfer to auditory‐leading pairs could simply be due to AV pairs not responding to any type of training. However, the lack of transfer to the visual leading‐pairs is not trivial, given that this condition is highly malleable and should profit from 2‐day AV training if sharing the same mechanism. This indicates that independent processes may indeed be at play in audiovisual temporal integration according to which sensory system is engaged first.

How do these behavioural findings integrate into current understanding of auditory and visual information processing and their interactions? Our evidence for separable processes driving perception of audiovisual simultaneity depending on leading sense suggests that temporal integration cannot easily be explained by a common multisensory nexus (e.g. in parietal cortex), but likely also involves processes at a more sensory level. This may be due to qualitative differences in how the visual and auditory system sample sensory signals and make use of information provided by other senses (van Wassenhove, 2013; Thorne & Debener, 2014; VanRullen *et al*., 2014). van Wassenhove (2013) put forth an interesting model of audiovisual integration in which fundamental differences in sampling strategies between audition and vision drive the asymmetries in the integration of AV vs. VA speech signals (i.e. the TBW asymmetries). In this view, predominantly auditory driven processes with high temporal resolution that play a role in phonemic encoding would contribute to the narrow AV window, while predominantly visually driven processes with slower temporal resolution that play a role in visemic encoding would contribute to the wider VA window. Another explanation for the AV–VA dichotomy may relate to how cues in one sensory modality may interact with distinct sampling mechanisms in the other modality, possibly involving crossmodal phase reset of brain oscillations (Lakatos *et al*., 2007, 2009; Kayser *et al*., 2008; Naue *et al*., 2011; Thorne *et al*., 2011; Romei *et al*., 2012; Mercier *et al*., 2013). Interestingly, it has been argued that, for audiovisual interactions, the nature of cross‐modal phase reset depends on the direction of the interaction, i.e. auditory‐to‐visual or visual‐to‐auditory (Thorne & Debener, 2014). Auditory‐to‐visual phase reset may serve to alert the visual system of imminent input (e.g. Thorne & Debener, 2014), which could be considered a low‐level (automatic) attentional mechanism potentially explaining the more efficient (i.e. narrower TBW) but less flexible (i.e. non‐trainable TBW) AV processing, reported in the present and previous work (Powers *et al*., 2009, 2012). In contrast, visual‐to‐auditory interactions is thought to be more driven by higher‐level prediction processes (see Thorne & Debener, 2014), flexibly adapting to changes in visual‐to‐auditory regularities (e.g., see Vroomen & Stekelenburg, 2010; Arnal & Giraud, 2012), which may be particularly relevant for speech processing where visual information has a predictive value for forthcoming auditory speech signals (Besle *et al*., 2008; Chandrasekaran *et al*., 2009). Such prediction‐based processes would be expected to span over a broader TBW and to be underpinned by more complex, flexible and feedback‐sensitive (i.e. trainable) neuronal circuits involving higher‐order multisensory convergence areas such as the superior temporal sulcus, which, notably, has been implicated in mediating the training benefit on the (visual‐leading) TBW (Powers *et al*., 2012). Whatever the mechanisms that could explain our results, our findings reveal that the narrower and less flexible TBW for auditory‐leading conditions vs. the broader and more flexible TBW for visual‐leading conditions may have their origin in auditory‐to‐visual and visual‐to‐auditory influences serving different functions.

In conclusion, we provide behavioural evidence that temporal audiovisual integration of a sound preceding visual input is dissociable from integration of a visual stimulus preceding a sound, and link this dichotomy to recent theories on cross‐modal influences on visual vs. auditory processing. Our data highlight the importance of considering that audiovisual temporal integration may not be a unitary process but depend on the leading sense. This is relevant when evaluating temporal integration in patient populations with presumed disorders in multisensory interactions (e.g., autism; Foss‐Feig *et al*., 2010), as selective impairment of AV and VA integration might relate to different aspects of cognition and require specific interventional approaches.

## Conflict of interest

The authors declare no competing financial interests.
